# Productivity in the Barents Sea - Response to Recent Climate Variability

**DOI:** 10.1371/journal.pone.0095273

**Published:** 2014-05-01

**Authors:** Padmini Dalpadado, Kevin R. Arrigo, Solfrid S. Hjøllo, Francisco Rey, Randi B. Ingvaldsen, Erik Sperfeld, Gert L. van Dijken, Leif C. Stige, Are Olsen, Geir Ottersen

**Affiliations:** 1 Institute of Marine Research (IMR), Bergen, Norway; 2 Department of Environmental Earth System Science, Stanford University, Stanford, California, United States of America; 3 Institute of Marine Research (IMR), Oslo, Norway; 4 Centre for Ecological and Evolutionary Synthesis (CEES), Department of Biosciences, University of Oslo, P. Blindern, Oslo, Norway; 5 Geophysical Institute, University of Bergen and Bjerknes Centre for Climate Research, Bergen, Norway; 6 Uni Climate, Uni Research AS and Bjerknes Centre for Climate Research, Bergen, Norway; University of Vigo, Spain

## Abstract

The temporal and spatial dynamics of primary and secondary biomass/production in the Barents Sea since the late 1990s are examined using remote sensing data, observations and a coupled physical-biological model. Field observations of mesozooplankton biomass, and chlorophyll *a* data from transects (different seasons) and large-scale surveys (autumn) were used for validation of the remote sensing products and modeling results. The validation showed that satellite data are well suited to study temporal and spatial dynamics of chlorophyll *a* in the Barents Sea and that the model is an essential tool for secondary production estimates. Temperature, open water area, chlorophyll *a*, and zooplankton biomass show large interannual variations in the Barents Sea. The climatic variability is strongest in the northern and eastern parts. The moderate increase in net primary production evident in this study is likely an ecosystem response to changes in climate during the same period. Increased open water area and duration of open water season, which are related to elevated temperatures, appear to be the key drivers of the changes in annual net primary production that has occurred in the northern and eastern areas of this ecosystem. The temporal and spatial variability in zooplankton biomass appears to be controlled largely by predation pressure. In the southeastern Barents Sea, statistically significant linkages were observed between chlorophyll *a* and zooplankton biomass, as well as between net primary production and fish biomass, indicating bottom-up trophic interactions in this region.

## Introduction

**Figure 1:**
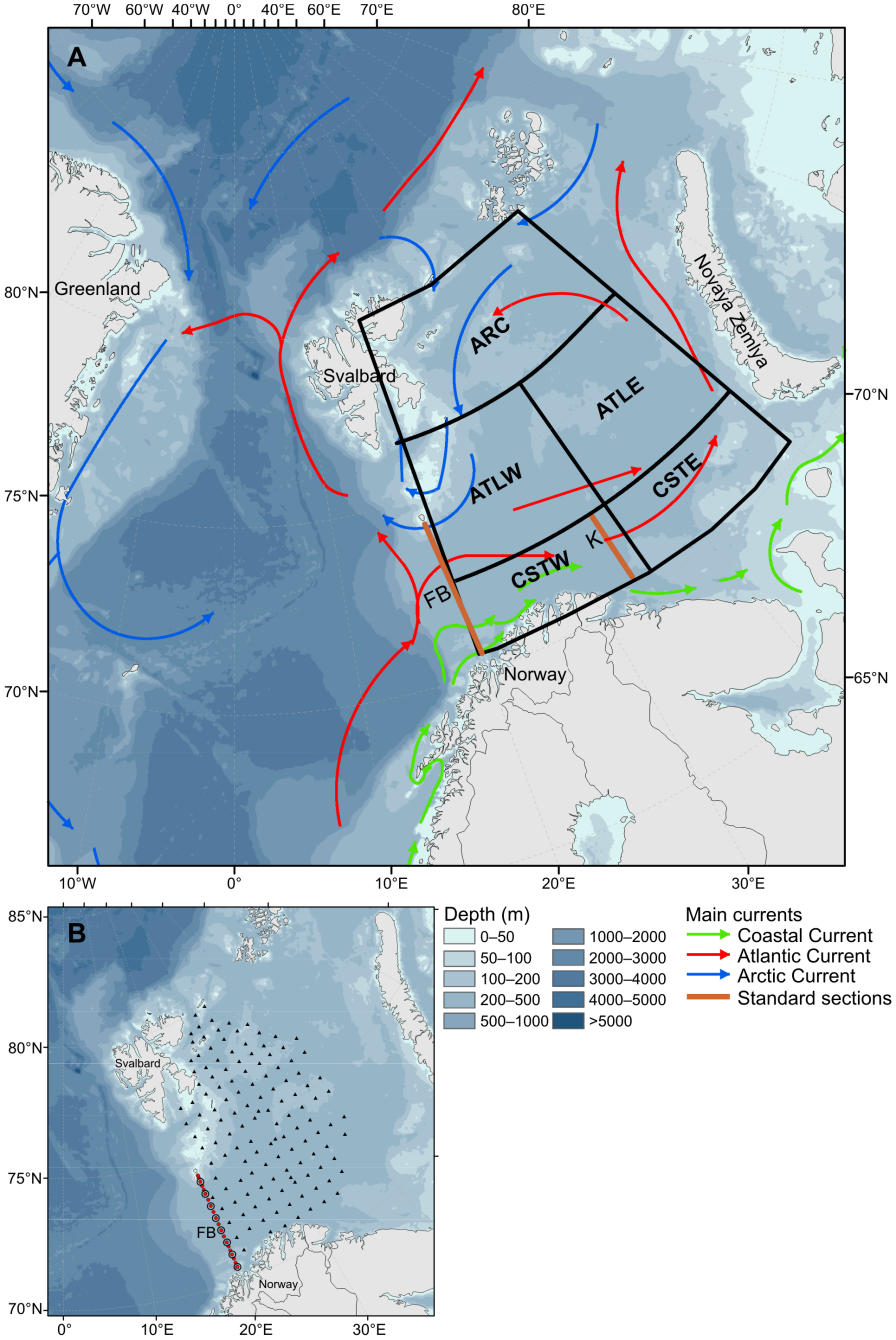
Overview of main currents and geographical range of the study area in the Barents Sea. (A) Warmer Atlantic water (red) meets colder Arctic water (blue). The Barents Sea was divided into five regions: CSTW = Coastal west, CSTE = Coastal east, ATLW = Atlantic west, ATLE = Atlantic east, ARC = Arctic (see Materials and Methods for more details). Red lines indicate sections within the Barents Sea: FB = Fugløya-Bjørnøya section, K = Kola; (B) Standard stations on FB section (Chl *a* = filled red circles; zooplankton = open circles) and autumn survey (August to early October) shown as an example for 2010 (triangles).

The Barents Sea is an open sub-Arctic shelf ecosystem situated north of Norway and north-west of Russia and ranging over latitudes from 68 to 82°N. It covers an area of 1.6 million km^2^ with an average depth of 230 m and connects with the Norwegian Sea to the west and the Arctic Ocean to the north. The physical and ecosystem dynamics of its coastal and southern regions are strongly influenced by the inflow of warm Atlantic water from the southwest, which causes large temperature fluctuations, especially in the western area [1] (Fig. 1A). The Barents Sea ecosystem supports some of the world’s biggest stocks of cod (*Gadus morhua*), capelin (*Mallotus villosus*) and haddock (*Melanogrammus aeglefinus*), and is the main nursery ground for the large stock of Norwegian spring spawning herring (*Clupea harengus*) [2], [3]. This ecosystem is also the home to one of the largest concentrations of seabirds in the world and a diverse assemblage of marine mammals [4]. There is a rich and diverse community of plankton in the system, sustaining these higher trophic levels [5].

**Figure 2:**
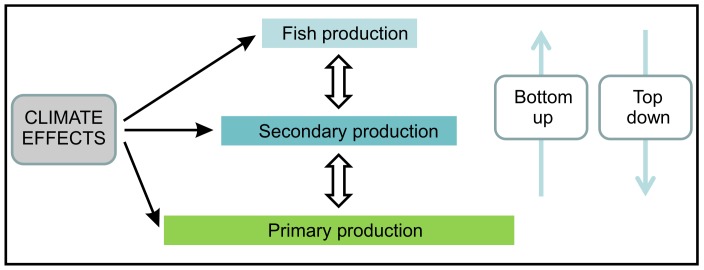
Schematic overview of potentially interacting ecosystem processes. Bottom-up and top-down effects on all trophic levels may interact with climate on all trophic levels to determine the biomass and production of phytoplankton, zooplankton and fish.

Zooplankton biomass in the Barents Sea seems to be controlled both by top-down (predation by fish) and bottom-up forcing (temperature, advection) [6]. Many previous ecosystem studies in the Barents Sea have focused on top-down control of zooplankton [7]–[9], exploring the predation pressure exerted by capelin, one of the key planktivorous fish species in this region. The fluctuations in zooplankton are inversely related to the fluctuations in capelin, and capelin stock size explains 40% of the interannual variation in total zooplankton biomass during the period 1984–2010 [5]. However, the energy flow from primary production through the food web ultimately limits upper trophic level fishery yields [10]. Bottom-up processes are therefore also important, in particular as changes in climate conditions (e.g. warming and reduced sea ice extent in the recent years) likely will influence the timing and magnitude of phytoplankton blooms and thus influence primary productivity of the ocean (Fig. 2). Based on their observations in the North Sea, Kirby et al. [11] pointed out the need to consider both top-down and bottom-up control to fully understand mechanisms of the marine ecosystems (cf. Fig. 2). Temporally varying degrees of top-down and bottom-up control have been found in the Barents Sea ecosystem [12].

The annual net primary production (NPP) in the Arctic Ocean (all waters north of the Arctic circle) increased yearly by an average of 27.5 Tg carbon (C) from 2003 to 2007 and by 35 Tg carbon (from 478 Tg C to 513 Tg C) between 2006 and 2007 [13]. Thirty percent of this increase is attributable to reduced summer ice areal extent and 70% to a longer phytoplankton growing season. Between 1998 and 2009, annual NPP in the Arctic Ocean increased by 20% [14]. These authors predict a continued increase in productivity as the sea ice declines further, which may alter marine ecosystem structure and the degree of pelagic-benthic coupling. Other studies predict that NPP may decrease globally in most areas due to increased stratification, except for in the Arctic Ocean where more open waters will allow greater production [15], [16]. A further increase in NPP will, however, also rely on greater nutrient availability in the Barents Sea, as we know that the short phytoplankton blooms in spring almost completely deplete nutrients, especially nitrate [17]. Recent studies also show that there are significant trends towards earlier phytoplankton blooms in the Arctic [18].

The key copepod species (*Calanus finmarchicus* and *C. glacialis*) as well as krill (*Thysanoessa inermis* and *T. raschii*) are regarded as predominantly herbivorous [19], [20]. They are in turn key food sources for the higher trophic level predators in the Barents Sea ecosystem. One can thus expect that changes in NPP will have significant impact on the biomass and production of zooplankton and hence fish.

In this study, we investigate how recent climate variability and change have affected seasonal and interannual variability in plankton biomass and productivity in the Barents Sea through bottom-up as well as top-down processes. Impacts on different seasons and regions are examined for 1998–2011, the period for which routine remotely sensed chlorophyll *a* (Chl) data are available. To obtain a comprehensive representation of primary and secondary productivity, we utilize a variety of data generated by a range of different methods, covering all or part of that period. These include: *in situ* observations, remote sensing data as well as modeling results. The links between NPP, zooplankton, and fish biomass time series are explored using correlation analyses. The response of these biological variables to climate effects was investigated and evaluated through the potentially driving physical variables temperature and open water area (area free of sea ice cover).

## Materials and Methods

### Ethic Statement

The authority who issued the permission for each location is the Institute of Marine Research (IMR), Bergen, Norway. This is a Norwegian governmental institute and the permission to conduct the study on this site was given by the Norwegian government. We confirm that the field studies did not involve endangered or protected species.

### Study Area

We divided the Barents Sea into three oceanographic sectors: Coastal (70.5–72.5°N, 20.0–50.0°E), Atlantic (72.5–76.5°N, 20.0–50.0°E), and Arctic (76.5–80.0°N, 20.0–50.0°E) waters. The Coastal and Atlantic waters were further split into two regions, one east and one west of 35°E (Fig. 1). We also analyzed data from the Fugløya-Bjørnøya (FB) section, which is located at the western entrance of the Barents Sea and surveyed up to 6 times each year.

### Temperature Data

Temperature data from the Kola section [21] (PINRO website. Available: http://www.pinro.ru/. Accessed 2013 August 5.) were used in interannual correlation analyses for the different Barents Sea regions. The temperature in the Kola section is quite representative for the Atlantic domain of the Barents Sea [22]. Further, the trends of the Kola section temperature show remarkable similarity to the Atlantic Multi-decadal Oscillation (AMO) index, demonstrating that the climate variation found in the Kola section is a local manifestation of larger-scale climate fluctuations covering the entire North Atlantic [23].

For a comparison of two years representing extreme climate conditions, surface temperature fields from 1998 and 2006 were derived using data collected during annual scientific surveys to the Barents Sea. For presentation purposes, the data have been interpolated onto a grid with 1/68 degree meridional resolution (18 km) and 1/28 degree zonal resolution (10–14 km).

### Remote Sensing Data - Sea Ice, Chlorophyll a and Open Water Area

Average April sea ice concentrations for the years 1998 and 2006 were estimated using Special Sensor Microwave/Imager (SSM/I) passive microwave remote sensing data from the National Snow and Ice Data Center (National Snow and Ice Data Center website. Available: http://www.nsidc.org. Accessed 2013 Aug 5.).

Daily NPP and open water area (OW) were calculated from satellite data as described in detail in Arrigo and Van Dijken [14]. Satellite-derived Chl *a* (Sat Chl *a*) was based on SeaWiFS retrievals for years 1998–2007 (SeaWiFS started to experience operational errors in 2008) and MODIS/Aqua data for years 2008–2011 (8 day level 3 binned, sensor default Chl algorithm) using the latest reprocessing (R2010.0 for SeaWiFS and R2012.0 for MODIS/Aqua).

For each of the five regions (Fig. 1A), mean NPP and OW was calculated on a daily basis. Spatially integrated NPP was calculated as the product of the mean NPP per unit area for a given region and the OW of that region. These values were integrated over each year to calculate the annual NPP of the whole area. Validation of Sat-derived Chl *a* estimates was restricted to the FB section as complete seasonal coverage of observed *in situ* Chl *a* data was only available for this section.

### *In situ* Chlorophyll a

Water samples for *in situ* chlorophyll *a* (Chl *a*) analyses were collected at standard depths (0, 5, 10, 20, 30, 50, 75, and 100 m). For each sample a volume of 263±2 ml of seawater was filtered through glass fiber filters (GF/C) and stored frozen until analysis ashore. At the laboratory, the pigments were extracted overnight in 90% acetone at 4°C. After centrifuging the extract, Chl *a* concentrations were measured by fluorometry using a Turner fluorometer (model 10AU) both before and after acidification with 5% hydrochloric acid [24].

Chl *a* data from the 20 stations (Fig. 1B) of the FB section from the period 1998–2011 were pooled and the mean concentrations for both the upper 20 and 50 m were calculated for each sampling date. Thereafter, monthly mean Chl *a* concentrations for each depth layer were calculated for comparison with Sat Chl *a* data and to examine seasonal dynamics.

### *In situ* Zooplankton

Mesozooplankton were collected with a WP2 plankton net along the FB section and during the regional and large-scale ecosystem cruises (Fig. 1B). The WP2 is a 0.56 m diameter plankton net with 180 µm mesh, towed vertically between the bottom and the surface. The FB section was sampled up to six times per year, covering all seasons, whereas ecosystem cruises were carried out in autumn (August to early October). The sampling of mesozooplankton was done at 8 of the 20 stations along the FB section, starting at 70°30′N and moving northwards at half degree intervals (Fig. 1B). The samples obtained were usually divided into two using a Motoda splitter. One-half was preserved in 4% formalin for analysis of species composition and abundance at the IMR laboratory. The other half was used for biomass estimation and was fractionated successively through three sieves: 2000 µm, 1000 µm, and 180 µm. The content of each sieve was rinsed briefly with freshwater to remove the salt and then transferred to pre-weighed aluminium trays. These were dried at 60°C for a minimum of 24 h and weighed to obtain dry weight biomass. For larger organisms, the drying period was prolonged until a constant weight was obtained. The results are expressed as dry weight biomass per m^2^ of water column, for the three size-fractions.

For comparison with model results (described beneath), we chose to use the sum of 2000–1000 µm and 1000–180 µm mesozooplankton fractions as a proxy for the *C. finmarchicus* biomass. We excluded the largest fraction (>2000 µm) as the amount of *C. finmarchicus* in this fraction was insignificant [25]. For the spatial comparison, we focused on August-September means, as these are the months with the best sampling coverage. We note that the proxy for *Calanus finmarchicus* biomass is constant and do not reflect time and spatial variations in *Calanus* species composition reported [26]. Thus, comparison of observed and modeled zooplankton data should be interpreted with care, especially in areas influenced by Arctic waters.

### Zooplankton Production Estimates from the Norwecom Model

We extended the studies of zooplankton biomass to zooplankton production by applying the modeling system norwecom.e2e [27]–[29], which consists of a full 3 dimensional ocean model and the biogeochemical model NORWECOM that is two-way coupled to an individual based model for *C. finmarchicus*. The model system has been validated and applied for the Norwegian Sea [27], [29] as well as applied recently for the Barents Sea (Skaret et al. unpublished). The two-way coupling allows both bottom-up and top-down processes to influence the abundance of *C. finmarchicus*. Predation from pelagic and meso-pelagic fish and invertebrates are included in the model as a spatial and size-dependent mortality rate on *C. finmarchicus* abundance. The model was run for the years 1998–2007 for the Norwegian and Barents Seas (see also Text S1, Fig. S1 & Fig. S2 for more details about the model and its validation).

### Pelagic Fish Stocks

Biomass of pelagic fish in the Barents Sea was extracted from the following reports and publications: age 1+ capelin from acoustic estimates in September [3]; age 1 and 2 herring from Virtual Population Analysis (VPA) estimates [30] using standard weights at age (9 g for age 1 and 20 g for age 2); age 1+ polar cod and blue whiting (*Micromesistius poutassou*) from acoustic estimates in September [31]; estimates of 0-group biomass of cod, haddock, and herring (were corrected for catch efficiency) [31], [32]. Biomass of 0-group fish was incorporated as these may impose considerable predation impact on mesozooplankton. The area for compiling fish biomass data varies somewhat from the defined study area and between the years.

### Data Analyses

To validate the remote sensing data, the Sat Chl *a* values were compared with observed Chl *a* at the FB section. Correlation analyses were performed using the monthly mean values of each year as well as monthly mean values averaged over all years. Further correlation analyses were performed to explore relationships between the time series of physical (Kola temperature, OW) and biological (NPP, Chl *a*, zooplankton biomass, capelin and pelagic fish biomass) variables. The strength of a correlation between two time series was estimated by the Pearson correlation coefficient (r) and significance was tested with consideration of autocorrelation in the two time series. To account for autocorrelation, the effective number of degrees of freedom (i.e. the number of independent joint observations, Nc, minus 2) in significance tests of correlations was adjusted following a method proposed by Quenouille [33] and modified by Pyper and Peterman [34]. Equations described by the above mentioned authors are summarized in a recent publication [5]. Trends in the physical and biological variables were described by Pearson correlation coefficients of the variables with time (year), without correcting for autocorrelation in significance tests.

## Results

### Climate Conditions

**Figure 3:**
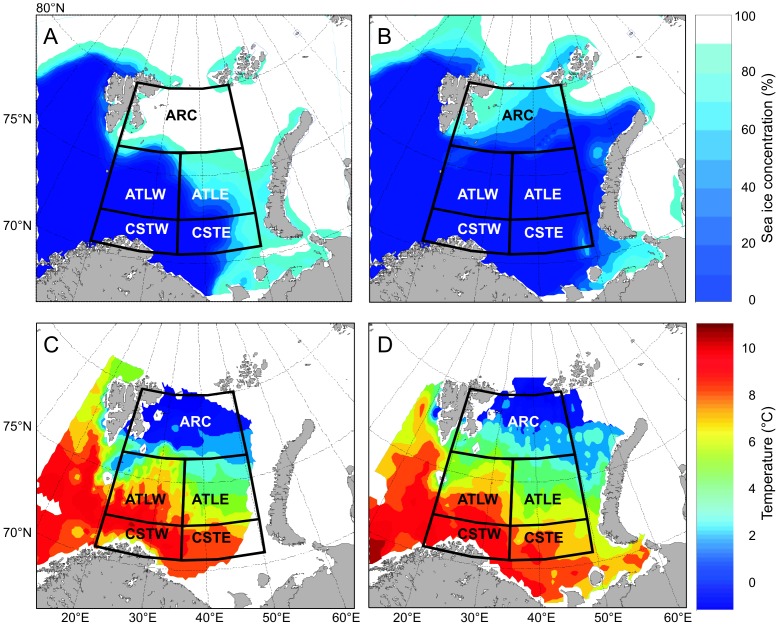
Example of extreme climate variability in the Barents Sea. Climate variability is illustrated as differences in (A, B) satellite derived sea ice concentration and (C, D) *in situ* surface temperature between the two extreme years 1998 (A, C) and 2006 (B, D).

**Figure 4:**
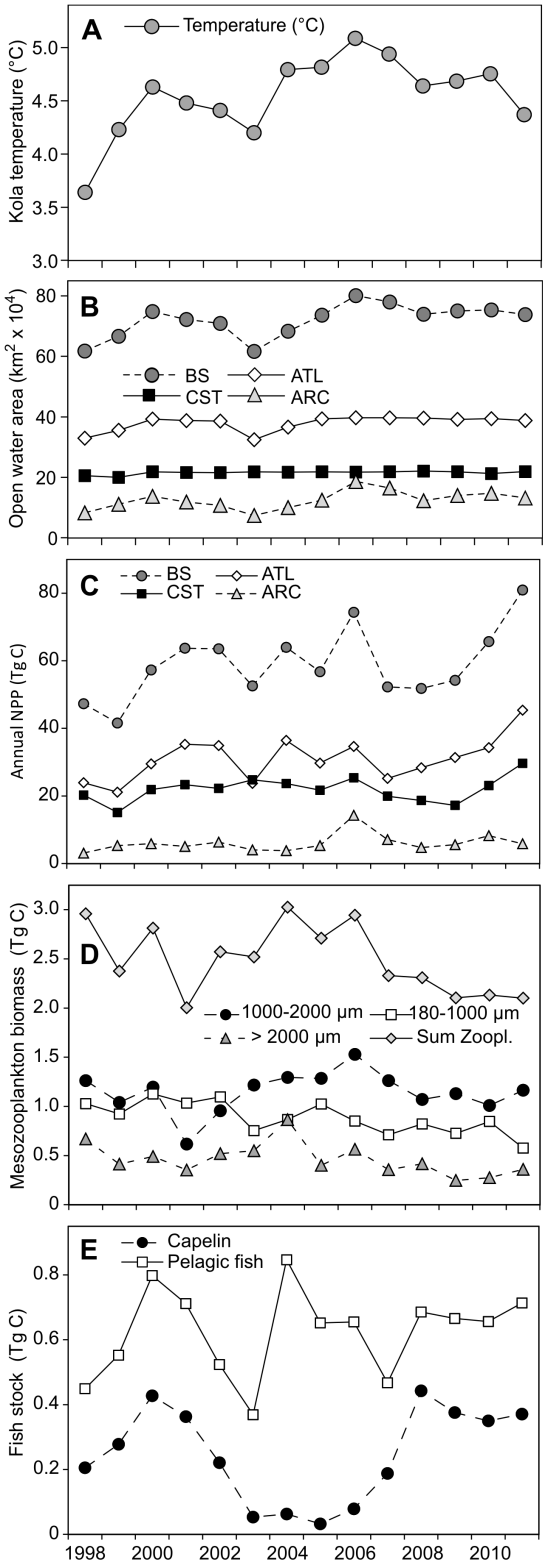
Time-series of abiotic and biotic variables during the study period 1998–2011. (A) Kola temperature, (B) mean open water area and (C) annual NPP for different regions of the Barents Sea, (D) mesozooplankton biomass of different size fractions and (E) fish stock for the Barents Sea study area. ATL = Atlantic sector (ATLE & ATLW), CST = coastal sector (CSTE & CSTW), ARC = Arctic region, BS = Barents Sea study area. For comparison among various trophic levels, original zooplankton dry weight (dw) and fish wet weight (ww) data were converted to carbon biomass (C) using a conversion factor of 43.5% C to dw for zooplankton ([71], all crustaceans) and a conversion factor of 10% C to ww for fish [72].

Over the study period 1998–2011, there have been strong interannual fluctuations in climate conditions (Fig. 3, Fig. 4A&B), which are likely to influence the NPP in the Barents Sea. Kola temperature as well as OW increased during the study period (Fig. 4A&B, Kola temp.: r = 0.56, t = 2.31, df = 12, p = 0.039, OW-BS: r = 0.61, t = 2.63, df = 12, p = 0.022). A comparison of sea ice concentration (April) and surface temperature (August-September) in the two most extreme years, 1998 and 2006 (Fig. 3), shows: (i) Arctic – a strong difference in winter sea ice extent and temperature, (ii) Atlantic west – very small differences in ice coverage and temperature, and (iii) Atlantic east – large difference in ice coverage (the ice completely retreated in the warm year, i.e. 2006). The coastal region of the Barents Sea, especially the western side, is normally not subjected to ice cover (Fig. 3B). However, moderate differences in temperature were observed between 1998 and 2006 in the coastal study area.

### Validation of Remotely Sensed Chl *a* Data

**Figure 5:**
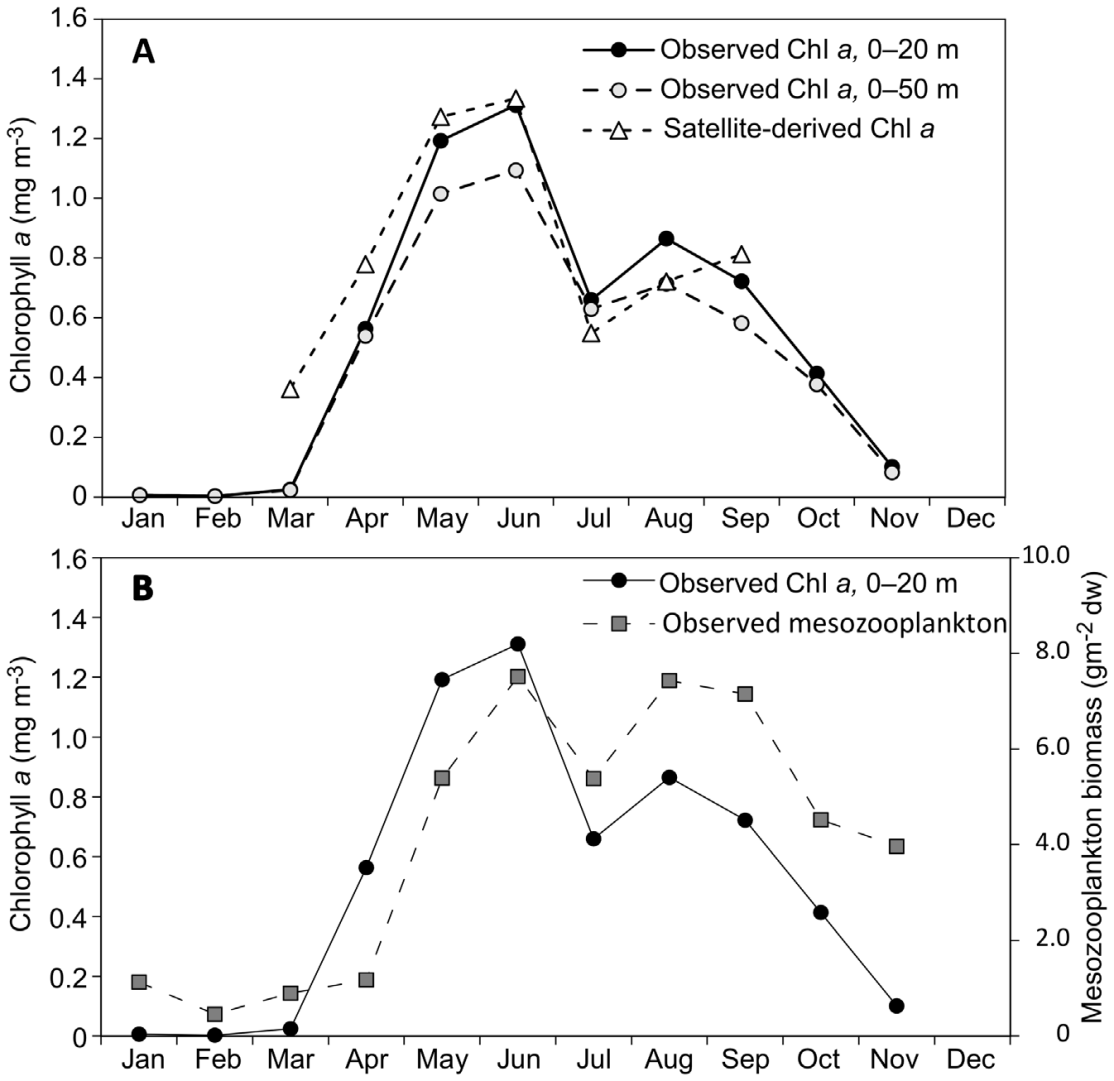
Seasonal dynamics of chlorophyll *a* (Chl *a*) and mesozooplankton. (A) Observed and satellite-derived Chl *a* concentrations and (B) observed Chl *a* concentration and observed mesozooplankton biomass (sum of 2000–1000 µm and 1000–180 µm as a proxy for *C. finmarchicus* biomass) from surface to bottom. Values are 1998–2011 averages for the FB section.

We used *in situ* Chl *a* data in the upper 20 m and 50 m to validate the time series of Sat Chl *a* concentrations at the FB section for the period 1998–2011. Our results for the monthly data averaged over all years at the FB section show that the seasonal dynamics and magnitude of the Sat Chl *a* concentrations are highly correlated with the observed Chl *a* concentrations both for the upper 20 m (Fig. 5A, r = 0.92, n = 7, Nc = 6.46, p = 0.0056, corrected for autocorrelation) and 50 m (Fig. 5A, r = 0.91, n = 7, Nc = 6.40, p = 0.0082, corrected for autocorrelation). We also found a highly significant relationship between Sat Chl a and observed Chl *a* concentrations using monthly mean values of each year during the entire study period (r = 0.69, n = 168, Nc = 16.81, p = 0.0023, corrected for autocorrelation).

The yearly means (pooled data March–September, 0–100 m) in observed and Sat Chl *a* concentrations tend to be positively correlated (r = 0.47, n = 14, Nc = 13.04, p = 0.105, corrected for autocorrelation), suggesting a match in the interannual variability. Moreover, the yearly values are of a similar order of magnitude (mean±SD, n = 14, observed Chl *a*: 0.72±0.20 mg m^−3^, Sat Chl *a*: 0.93±0.28 mg m^−3^). The Sat Chl *a* values, however, are somewhat higher than the observations (two-sided t-test: t = 2.34, p = 0.028). This mostly originates from a few extraordinarily high satellite-derived values (corresponding to >7 mg m^−3^) from the coastal areas. Excluding data >7 mg Chl *a* m^−3^ brings the satellite data (mean±SD, n = 14, observed Chl *a* <7 mg m^−3^∶ 0.83±0.19 mg m^−3^) closer to observations (two-sided t-test: t = 1.57, p = 0.129). The results indicate that the remotely sensed Chl *a* data reproduce *in situ* observations well in the Barents Sea opening, and thus are well suited to study temporal and spatial dynamics of Chl *a* and thus NPP in the Barents Sea.

### NPP in Different Regions

The mean annual NPP for the Barents Sea is 59.0 Tg C year^−1^ (Table 1) with the lowest rate (41.6 Tg C year^−1^) in 1999 and the highest (80.9 Tg C year^−1^) in 2011. The largest contribution to the mean annual NPP in the Barents Sea comes from the Atlantic region (53%), followed by the coastal (37%) and the Arctic (10%). However, considering the production per unit area for the whole study period, the coastal values are higher than the Atlantic and Arctic (Table 1).

**Table 1:** Mean satellite-derived chlorophyll *a* (Sat Chl *a*), net primary production (NPP) per unit area, annual NPP, and open water area (OW) for different regions of the Barents Sea during the study period.

Region	Sat Chl *a* (mg m^−3^)	NPP per area (g C m^−2^ year^−1^)	Annual NPP (Tg C year^−1^)	OW (km^2^)
CSTW	1.24±0.29 (23)	113.4±18.1 (16)	11.3±1.8 (16)	100039
CSTE	1.42±0.42 (29)	92.8±19.1 (21)	10.6±2.3 (22)	115274±5690 (5)
ATLW	1.23±0.35 (29)	90.4±16.8 (19)	17.3±3.3 (19)	191343±7589 (4)
ATLE	1.34±0.42 (32)	77.2±13.9 (18)	13.7±3.7 (27)	187089±18907 (10)
ARC	0.75±0.27 (36)	44.1±10.8 (24)	6.1±2.7 (45)	124805±30177 (24)
BS	1.26±0.28 (22)	92.4±14.0 (15)	59.0±10.5 (18)	718551±55085 (8)

Means ± SD and coefficient of variation (%) in parentheses are given for the yearly means during the study period (1998–2011, n = 14). CSTW = Coastal west; CSTE = Coastal east; ATLW = Atlantic west; ATLE = Atlantic east, ARC = Arctic and BS = Barents Sea study area. For more details of area definitions see Fig. 1. CSTW is always ice-free, i.e. there is no variability in OW.

The satellite-derived annual NPP in the Barents Sea increased nearly significantly during the study period (Fig. 4C; r = 0.52, t = 2.10, df = 12, p = 0.058). The interannual variation of NPP in the Atlantic sector covaries well with the NPP of the whole study area (r = 0.93, n = 14, Nc = 10.19, p<0.0001, corrected for autocorrelation), not surprisingly as this sector is the largest contributor to the total NPP (Fig. 4C, Table 1). The highest annual NPP for the Atlantic and coastal sector was observed in 2011, amounting to 45 and 30 Tg C, respectively (Fig. 4C).

**Figure 6:**
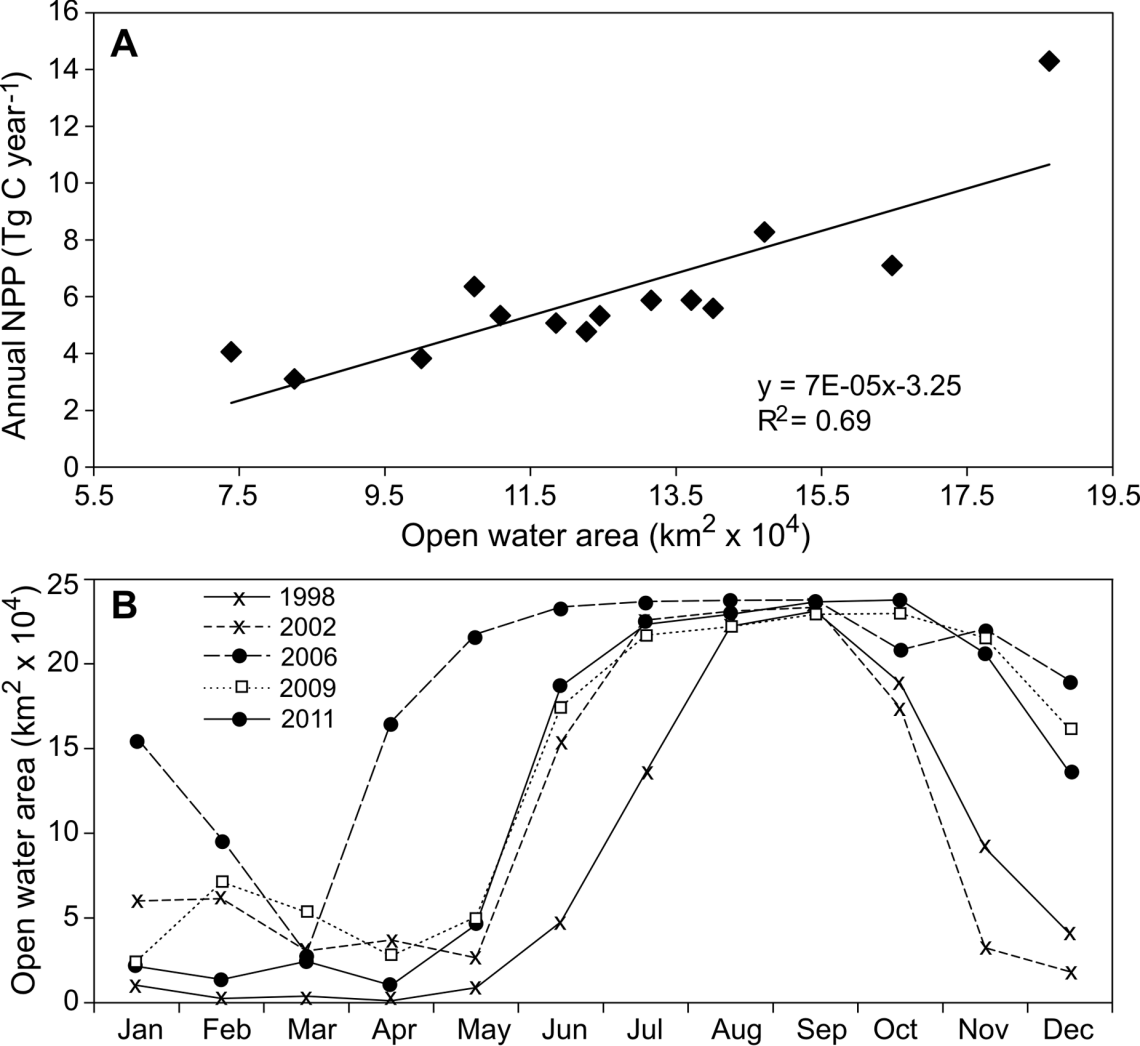
Annual net primary production (NPP) in the Arctic region of the Barents Sea. (A) Annual NPP in relationship to the mean open water area. (B) Seasonal development of open water area for five different years.

Compared to the Atlantic and coastal sectors, the Arctic sector contributed less to total NPP but showed a larger interannual variability (Fig. 4C; Table 1, see coefficient of variation), with the highest NPP observed in 2006 (14.3 Tg C year^−1^) and the lowest in 1998 (3.1 Tg C year^−1^). We also observed a positive relationship between OW and annual NPP in the eastern Atlantic region (r = 0.70, n = 14, Nc = 11.49, p = 0.014, corrected for autocorrelation) and even stronger in the Arctic region (Fig. 6A, r = 0.83, n = 14, Nc = 11.28, p = 0.001, corrected for autocorrelation). In addition, the onset and thus the duration of the open water season in the Arctic region also varied greatly between years (Fig. 6B). The year 2006 showed the longest open water season, with the peak extending from May to December, likely contributing to the high NPP in that year. In contrast, there was a very short open water season in 1998, lasting from July to October, which probably resulted in the very low NPP.

### Chl *a* and Mesozooplankton Dynamics

The time series of *in situ* and Sat Chl *a* over 1998–2011 were used to examine spatial patterns and timing of the annual phytoplankton bloom maximum. Pooled *in situ* Chl *a* data for the study period from the FB section show that the peak in productivity (∼peak in the seasonal cycle of Chl *a* at 1.3 mg m^−3^) occurs in May–June (Fig. 5A).

**Figure 7:**
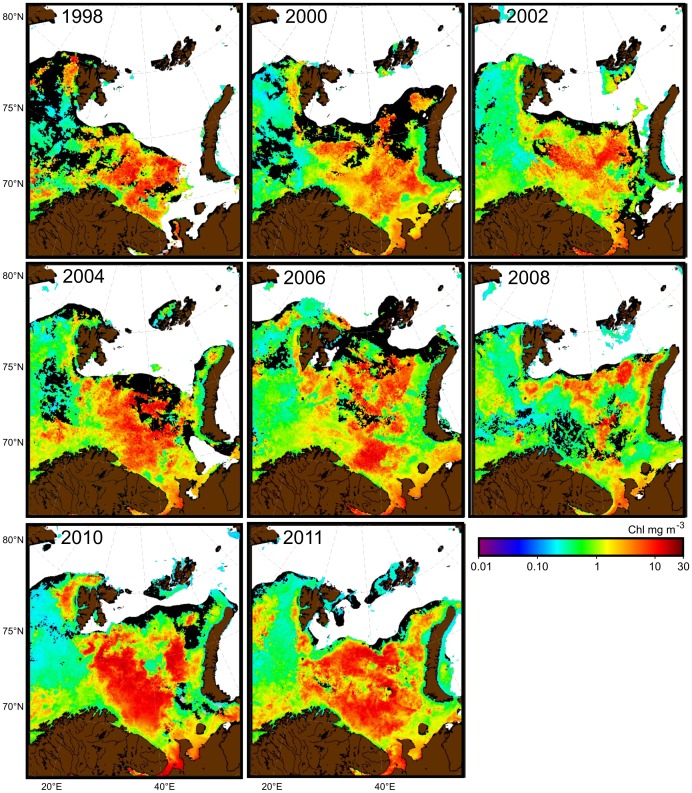
Satellite-derived surface chlorophyll *a* (Chl *a*) concentrations (mean for May) in the Barents Sea. White indicates ice cover; black shows missing values.

Examination of large-scale distribution patterns from remote sensing data also shows that the Sat Chl *a* concentrations peak in May and that there are large interannual variations in spatial patterns (Fig. 7). The most striking trend is the northward and eastward extension of the phytoplankton bloom in May, especially evident after 2004 (Fig. 7), probably due to the larger sea ice retreat that has occurred in recent summers.

The seasonal dynamics of *in situ* Chl *a* and *C. finmarchicus* biomass at the FB section show that the development of zooplankton starts with a lag time of one month after the initiation of the phytoplankton bloom, and that maximum biomass occurs from June through September (Fig. 5B). Correspondingly, the strength of the relationship between monthly *in situ* Chl *a* and zooplankton biomass increased when Chl *a* data were lagged by one month (r = 0.86, n = 16, Nc = 9.97, p = 0.0015, corrected for autocorrelation) compared to no lag time (r = 0.73, n = 74, Nc = 19.96, p = 0.0002, corrected for autocorrelation).

### Observed Mesozooplankton Biomass

The mean zooplankton biomass observed on the fall ecosystem surveys for the study period was generally high in the coastal and Atlantic west (>7.2 g m^−2^, Table 2), followed by the Arctic (5.98 g m^−2^) and Atlantic east (5.37 g m^−2^). The medium size fraction (1000–2000 µm), which is assumed to consist largely of *C. finmarchicus*, contributed most to the zooplankton biomass, followed by the smallest size fraction (Table 2), which is assumed to contain less of *C. finmarchicus*. The eastern coastal region generally showed the strongest interannual variability (Table 2), which might be attributable due to low data availability or changes in hydrographical conditions.

**Table 2:** Mean mesozooplankton biomass for three size fractions and different regions of the Barents Sea during the study period (1998–2011).

Region	n	>2000 µm (g m^−2^ dw)	2000–1000 µm (g m^−2^ dw)	1000–180 µm (g m^−2^ dw)	Sum (g m^−2^ dw)
CSTW	263	0.85±0.55 (65)	3.80±1.23 (32)	2.59±0.94 (36)	7.24±1.98 (27)
CSTE	25	1.63±2.51 (154)	3.60±1.41 (39)	2.62±0.87 (33)	7.85±3.99 (51)
ATLW	647	1.56±0.57 (37)	3.56±0.62 (17)	2.44±0.55 (22)	7.56±1.18 (16)
ATLE	318	0.86±1.38 (161)	2.10±0.13 (6)	2.41±0.05 (2)	5.37±1.42 (26)
ARC	401	1.28±0.68 (53)	2.61±1.34 (51)	2.09±0.63 (30)	5.98±2.10 (35)
BS	1654	1.25±0.44 (35)	3.09±0.56 (18)	2.38±0.44 (18)	6.71±0.95 (14)

Means ± SD and coefficient of variation (%) in parentheses are given for observed mesozooplankton biomass (gm^−2^ dw). Abbreviations of regions as in Table 1.

Time series analyses showed large interannual variation in zooplankton biomass for the three different size fractions integrated over the entire Barents Sea (Fig. 4D). The biomass of the medium size fraction (2000–1000 µm) was highest in 2006 (1.53 Tg C, ∼4.1 g m^−2^ dry wt), corresponding to a year with the highest Kola temperature, OW and relatively high NPP. Low capelin stock size (0.08 Tg C, ∼0.8 million tonnes wet wt) was observed in 2006 as in the three preceding years, suggesting that the level of predation pressure may not be the only cause for the zooplankton biomass increase in 2006. The biomass of the medium size fraction (dominated generally by *C. finmarchicus*) remained stable (∼1.1 Tg C, ∼3 g m^−2^ dry wt) during 2008–2011, despite increasing NPP. However, this period was characterized by high predation pressure from pelagic fish, e.g. capelin (Fig. 4E). The interannual dynamics were probably driven by the combination of bottom-up and top-down factors throughout the period. A general decrease in zooplankton biomass was observed for the largest size fraction since 2004 (r = −0.74, t = 2.72, df = 6, p = 0.035), and for the smallest size fraction during the entire study period (r = −0.75, t = 3.95, df = 12, p = 0.002).

### Modeled Zooplankton Production

**Figure 8:**
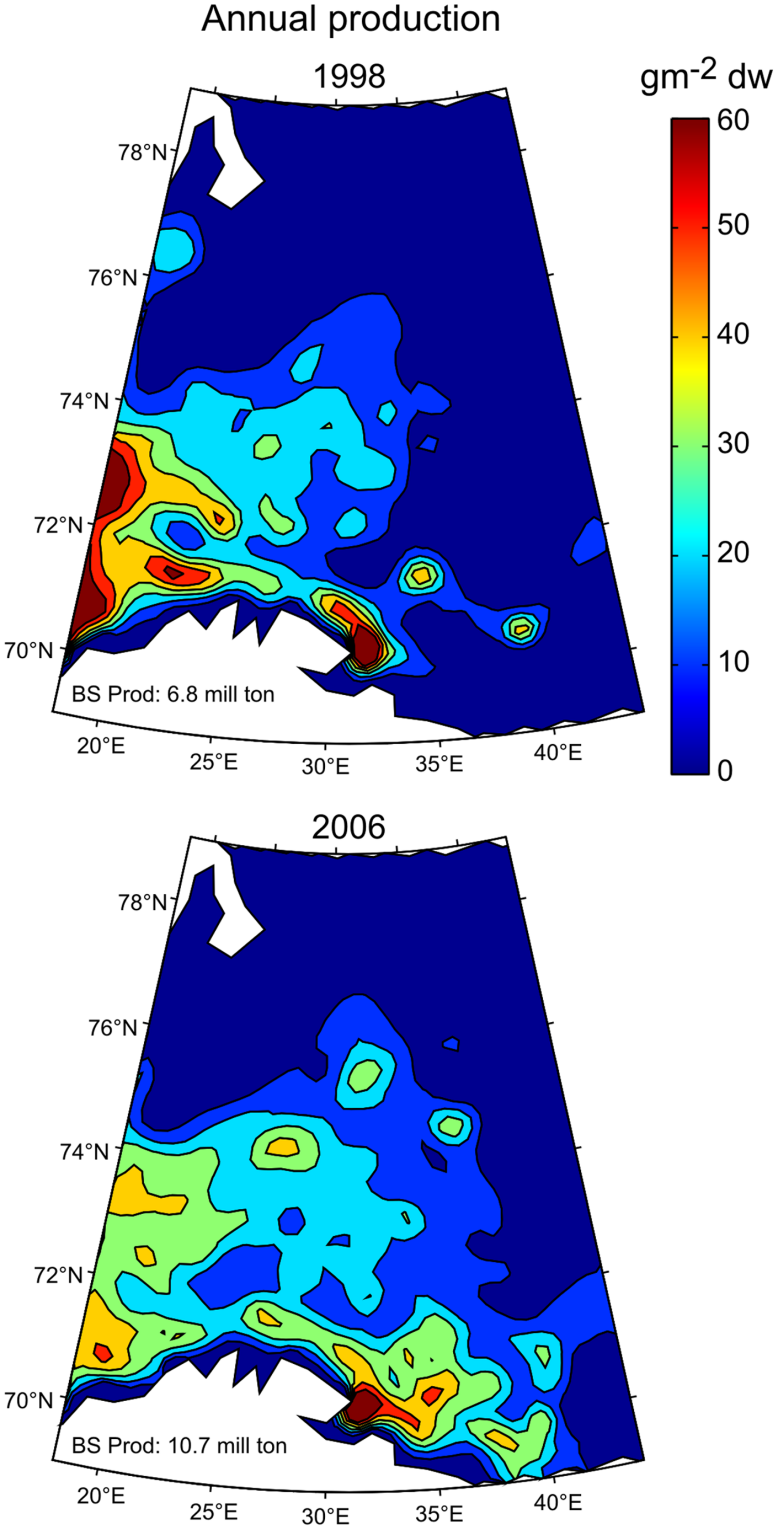
Modeled annual production (g m^−2^ dw) of *C. finmarchicus* in two selected years (1998 and 2006). The total annual *C. finmarchicus* production (in million tonnes) within the whole BS area is stated in the lower left of the figure (BS Prod).

Zooplankton production is difficult to measure, but can be obtained by using production to biomass (P/B) ratios from literature or modeling results, in this case the norvecom.e2e model. Even though, the model biomass values deviate somewhat from the observed ones (Fig. S2), the model captures the main dynamics and thus can provide production estimates with a spatial resolution not possible with other methods. The distribution patterns for the two example years show that the area of high secondary production extended to the south eastern areas of the BS in 2006 compared to 1998 (Fig. 8). The average secondary production for the whole BS region was much higher in 2006 (∼11 million tonnes dry wt.), than in 1998 (∼7 million tonnes dry wt.), though the modeled biomass values were quite similar in these years (Fig. S2). The temperature and NPP in the BS was much higher in 2006 compared to 1998, which could explain partly the higher secondary productivity in 2006.

### Relationships between Climatic & Biotic Variables

**Figure 9:**
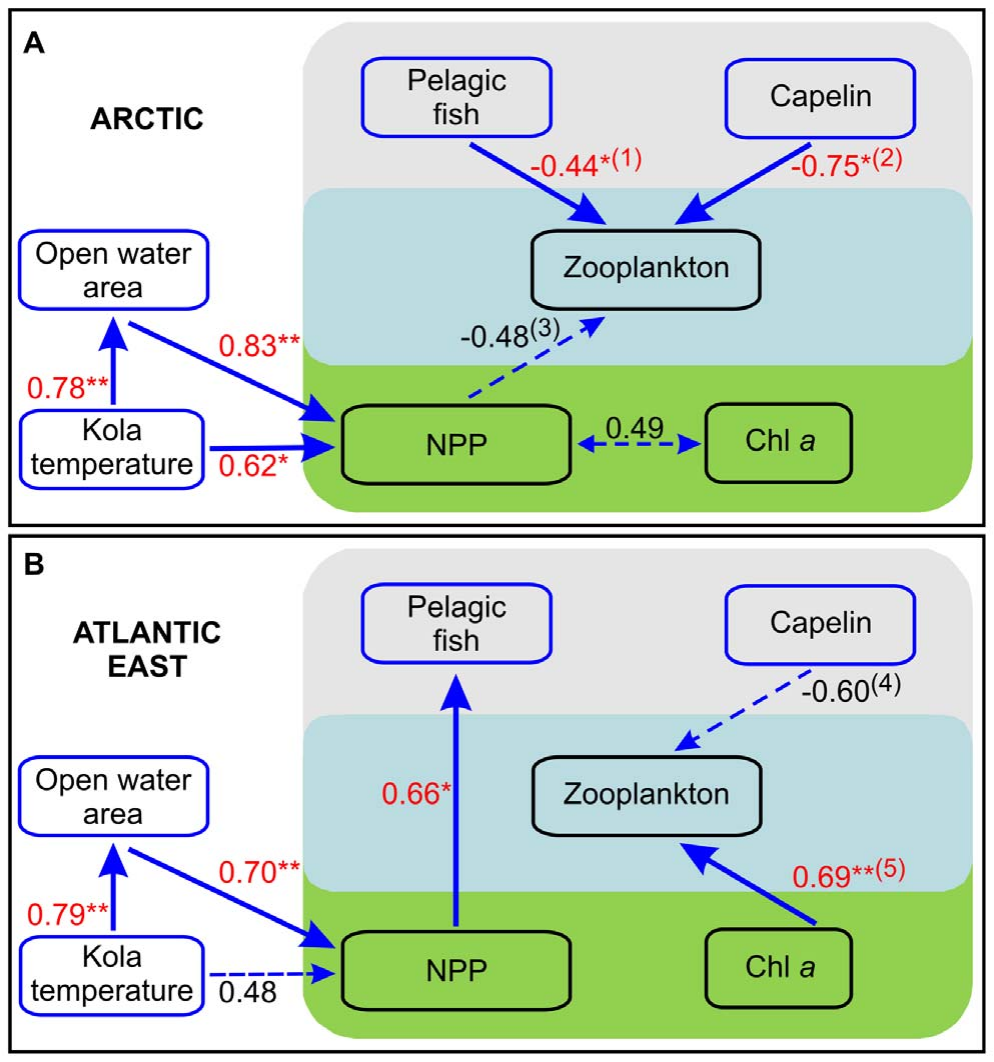
Relationships among variables in the Arctic and eastern Atlantic region of the Barents Sea. Red indicates significant Pearson correlation coefficients taking autocorrelation into account (*** p<0.001, ** p<0.01, * p< = 0.05; coefficients <0.4 are not shown, see also Supporting information Table S2&3). (1) Pelagic fish vs. zooplankton 180–1000 µm; (2) capelin vs. sum zooplankton (or capelin vs. zooplankton 1000–2000 µm, r = –0.79*); (3) NPP vs. zooplankton 180–1000 µm; (4) capelin vs. zooplankton 180–1000 µm (or capelin vs. zooplankton >2000 µm, r = –0.54); (5) Chl *a* vs. sum zooplankton (or Chl *a* vs. zooplankton >2000 µm, r = 0.72**).

In order to reveal any linkages between climate, plankton, and fish for the different Barents Sea regions, associations among variables were quantified using Pearson correlations (see Tables S1–S6) and summarized for potentially important and meaningful interrelationships (Table 3). Significant relationships among variables for the Arctic and eastern Atlantic region of the Barents Sea were clearly evident (Table 3, Tables S1–S6), corroborating the findings of our other spatial analyses (cf. Fig. 3, 6, 7). There are almost no significant relationships between climatic and biotic variables for the Atlantic west and coastal areas of the Barents Sea (Table 3). The temperature of the Kola section is significantly correlated to the OW in almost all regions. The NPP is positively correlated to Kola temperature and OW in the Arctic, but significantly only to OW area in the Atlantic east (Table 3, Fig. 9). We found no relationship between NPP and zooplankton biomass, but a strong positive relationship between annual mean Chl *a* and zooplankton biomass in the Atlantic east (Fig. 9B). Our investigations show negative correlations between biomass of capelin and total pelagic fish with various size fractions of zooplankton in the Atlantic east and especially in the Arctic, indicating a high feeding pressure of fish on zooplankton in those areas (Fig. 9).

**Table 3:** Overview of correlation analyses examining relationships between various climatic and biotic variables in different regions of the Barents Sea.

Relationship	ARC	ATLE	ATLW	CSTE	CSTW	BS
Kola temperature – Open water area	**/**	**/**	**/**	(**/**)	–	**/**
Kola temperature – NPP	**/**	–	–	–	–	–
Open water – NPP	**/**	**/**	–	–	–	–
Sat Chl *a* – Zooplankton biomass	–	**/** ^(1)^	–	–	–	–
Fish/Capelin – Zooplankton biomass	**\** ^(2)^	(**\**)^(3)^	–	–	–	–
NPP – Fish biomass	–	**/**	–	–	–	–
Time – NPP	–	**/**	–	–	–	–
Time – Zooplankton biomass	**\** ^(4)^	**\** ^(5)^	–	–	–	**\** ^(6)^
Time – Fish/Capelin biomass	–	–	–	–	–	–

Abbreviations of regions as in Table 1. Relationship between variables: / positive significant (p<0.05), \ negative significant (p<0.05), – not significant, lines in parentheses p<0.1 (see Supporting information Table S1–S6 for underlying Pearson correlation coefficients taking autocorrelation into account). Further specifications of the used variables are indicated by superscript numbers in parentheses. (1) zooplankton >2000 µm and also sum zooplankton biomass; (2) capelin biomass vs. zooplankton 1000–2000 µm and vs. sum zooplankton biomass, total pelagic fish biomass vs. zooplankton 180–1000 µm (3) capelin biomass vs. zooplankton >1000 µm; (4) zooplankton >2000 µm and also 180–1000 µm; (5) zooplankton 180–1000 µm and also sum zooplankton biomass; (6) zooplankton 180–1000 µm.

## Discussion

### Climate Variability

The Barents Sea has experienced significant warming in recent years. The observed temperature variability in the Barents Sea is substantial and since the early 1970s there has been a long-term temperature increase in its southern region of almost 1.5°C [12], [22]. Correspondingly, there has been an increase in oceanic heat transport and a strong (50%) retreat of the Barents Sea winter sea ice [35]. The Barents Sea has been warmer in the last decade than ever before observed in the 110 years of observations [36].

The flow of Atlantic water into the BS is undoubtedly linked to atmospheric conditions [37], but the exact nature of the relationship is not clear. The North Atlantic Oscillation (NAO), which is the leading pattern of extra-tropical atmospheric variability over the north Atlantic, was correlated with the amount and temperature of the Atlantic water flowing into the Barents Sea, as well as sea ice cover up until the mid/late 1990s, e.g. [12], [38]. However, strong heat transport into the Barents Sea continued after this period due to atmospheric structures not captured by the large-scale NAO pattern. Thus, the inflow of warm Atlantic water to the Barents Sea has likely increased steadily since the late 1970’s and has remained high in recent years [35]. This heat has also been transferred to the Arctic waters in the northern Barents Sea. After the millennium shift, the variability and temperature in the Arctic waters has increased and the ice extent decreased [5], [39].

Although the years we analyse cover a relatively short period for investigating climate effects, there are strong indications of striking climatic changes in the recent years, e.g. elevated temperature conditions, drastic sea ice decrease, increased open water area and prolonged duration of the open water season. These climate patterns are also evident in longer-term studies [5], [12], [39]. Our analyses clearly reveal that climate effects during the study period are most notable in the Arctic and eastern Atlantic parts of the Barents Sea.

### Chlorophyll a and NPP Dynamics

Comparison of Sat Chl *a* with *in situ* Chl *a* and NPP data show that satellite-derived data are well suited for investigating seasonal and temporal dynamics of NPP in the Barents Sea. The results from this study reveal that satellite-derived NPP estimates are similar in magnitude to conventionally derived estimates [17]. Examination of the spatial pattern of Sat Chl *a* concentrations on an ecosystem scale show that the maxima generally occur in May, and that there is large interannual variability in the range and spatial distribution patterns of Chl *a* concentrations.

The annual cycle of NPP in the Barents Sea is typical for high latitude regions with a pronounced phytoplankton spring bloom fuelled mainly by winter regenerated nutrients. This spring bloom, that normally peaks in May, constitutes about half of the annual NPP and is the basis for most of the secondary production in the Barents Sea [17]. The magnitude of the annual NPP is strongly dependent on the availability of nutrients [17], [40]. In spring, this availability is determined by winter nitrate concentrations, resulting in high rates of “new production”. Later in the year, after stratification, most of the production is based on regenerated nutrients (e.g. ammonium). In certain areas, however, and due to strong wind induced mixing of deep-water nitrate into surface waters, new production can be important in this period as well.

Winter nutrients in the Atlantic waters of the Barents Sea have declined during the period of our study [41]. Silicate concentrations have declined by about 20% and nitrate by about 7% [41]. This decline in winter nutrients is likely associated with the increasing advection of warmer Atlantic waters into the Barents Sea in the last two decades [35]. The influence of Atlantic waters which originates much further south in the North Atlantic, is caused by large atmospheric changes [42], [43].

The observed Chl *a* and zooplankton biomass strongly co-vary seasonally, which fits well with previously reported seasonal patterns of the Barents Sea [17]. The spring/early summer peak of zooplankton lags that of phytoplankton by a month, similar to previous findings from the Barents Sea [44] and other regions [45]. The only region where we found a significant positive relationship between the interannual variability in zooplankton biomass and Chl *a* is the eastern Atlantic sector.

A review on the influence of climate variability in the Barents Sea concludes that there has been an increase in NPP during the recent warming [46]. This conclusion is based on model studies of primary production for the period 1981–2004, and a comparison with observations from other regions (e.g. Eastern Bering Sea) experiencing similar rising annual temperatures as well as decreasing sea ice extent. Our analysis of the NPP time series based on satellite-derived data similarly indicates that NPP in the Barents Sea ecosystem has increased moderately over the years (1998–2011), likely influencing the production at higher trophic levels.

Variability in annual NPP is relatively high in the Arctic region, compared to Atlantic and coastal regions. The Arctic region is subjected to much larger changes in sea ice cover. The decline in sea ice extent observed in recent years and the larger open water area enables light penetration and stabilization of the water column, which probably contributed to the higher NPP. The longer open water season in warm years has probably also contributed to an increase in NPP. However, the magnitude of increase in the annual NPP is smaller in the Barents Sea ecosystem when comparing with the Arctic Ocean, which includes all waters north of the Arctic Circle [14], [47]. This is because the contribution of the Arctic sector to the NPP of the whole Barents Sea study area is relatively small compared to the contribution of the Atlantic and coastal sectors.

Another aspect of NPP is the production occurring under the ice, which is not included in our analysis. A recent study indicates that there is more phytoplankton under Arctic sea ice than previously thought (at least in the Chukchi Sea), and that the NPP estimates in the Arctic Ocean are even higher when the ice associated production is included [48]. Large blooms under sea ice have also been reported for the Barents Sea region [49]. Ice associated crustaceans generally spend their entire life on the underside of the Arctic sea ice [50]. Although we do not consider the production under ice in this study due to lack of data, we are aware of its importance to ice (sympagic) and associated fauna [20], [50], [51]. In addition, ocean acidification may change phytoplankton species composition and increase NPP due to increasing concentrations of CO_2_ in surface seawater [52], [53]. Ocean acidification, however, is a slow process compared to the drastic changes in sea ice cover and temperature that can occur in the Barents Sea. Therefore, the potential effect of acidification on phytoplankton composition and NPP might be in the short term much smaller than the effects of sea ice retreat and warming in the ecosystem.

### Zooplankton Dynamics and Trophic Linkages

Over the study period, there have been considerable changes in zooplankton biomass. Time series analyses revealed that the biomass of particular size fractions of zooplankton decreased in the Arctic (the smallest and the largest size fraction) and Atlantic east (only the smallest size fraction, see Table 3). Further examinations of the links between zooplankton and fish biomass suggest that especially the northern region of the Barents Sea experienced significant predation pressure from capelin. Previously published long-term studies from the Barents Sea also show that year-to-year changes in zooplankton biomass appear to be strongly controlled by capelin [5], [9].

Results from this study show that modeled *C. finmarchicus* biomass data from the FB section and the Barents Sea study area reproduce main spatial and seasonal patterns, although an overestimation in autumn biomass is evident. This overestimation may be a model artefact (e.g. too high *C. finmarchicus* growth or too low predation) or an effect of overly strong advective import of *C. finmarchicus* (due to an inherent circulation model). Previously published studies revealed that advected biomass is important for maintaining high zooplankton production levels in the Barents Sea [5], [54]–[57].

The modeled *C. finmarchicus* biomass and production show large interannual variation in their spatial patterns. These variations may reflect varying climatic conditions, for example, temperature, ice extent or water volume transport (advection), but also feeding conditions such as the magnitude of NPP or predation. Fish predation has been parameterized as a function of copepod size and distribution in the model, and thus may not represent interannual variability in observed fish biomass. The estimates of production are fundamental to study food web dynamics in the Barents Sea both under present and future climate conditions. Thus more focus on improving and exploring model results, as a useful tool for future predictions of production estimates, will be a priority in upcoming research.

If we assume a P/B ratio of 6 for zooplankton [25] and 1.5 for pelagic fish [58], we obtain average trophic transfer efficiencies of 26% (min–max: 16–38) and 6% (min–max: 4–9), respectively from phytoplankton to zooplankton and from zooplankton to fish (based on Fig. 4) for the study period. The trophic transfer efficiency values may change depending on the P/B ratios used. Our results indicate a high transfer efficiency to zooplankton (>20%) and somewhat lower to fish (<10%). Nevertheless, these values are in the range of possible values reported for the North Atlantic [25]. Correlation analyses between physical and biotic variables indicate that the NPP in the Atlantic region of the Barents Sea, especially in the eastern region, influences fish biomass. Studies from other regions also show an association of high fish productivity with high Chl *a* and NPP [10], [59]. The fish biomass in the Barents Sea is currently at a very high level, probably supported by the relatively stable productivity of plankton. In recent years, the spatial distribution of capelin and cod has extended further north (∼82°N) and east than previously observed [3], indicating good feeding and growth conditions in these regions of the Barents Sea.

### Top-down versus Bottom-up Interactions

As stated above, many previously published studies have shown that top-down control of zooplankton is a key process potentially regulating zooplankton biomass and hence production in the Barents Sea. However, a model study has shown that a reduction in overwintering biomass of zooplankton, may not necessarily influence the production in the following years [60]. In their study, the results differed between the two main *Calanus* species: while the overwintering biomass of *C. finmarchicus* did influence the next year’s production of this species, the opposite was seen for its Arctic counterpart *C. glacialis*. Our results showing negative correlations between the biomass of pelagic fish and some zooplankton size fractions, indicate a top-down control of fish on zooplankton in the Atlantic east and particularly the Arctic region. This is in accordance with findings of another study based on long term data series of combined Soviet-Russian (1959–1990) and Norwegian (1984–2010) sources [9]. By means of statistical modeling, these authors found that fish predation explained >50% of the interannual variability in the biomass of medium sized and large mesozooplankton in the northern and central Barents Sea, and that the predation effect remained statistically significant when accounting for climate effects.

The question of predation as a regulator of zooplankton is disputed, as exemplified by the discussion regarding the drivers of the dynamics of northwest Atlantic continental shelf ecosystems. It has been proposed that while top-down forcing can be important at higher trophic levels in many northwest Atlantic shelf ecosystems, its impacts on zooplankton, phytoplankton, and nutrients are minor or nonexistent [61], [62]. Instead, these contributions state that lower trophic-level dynamics in these ecosystems are governed by climate-associated bottom-up forcing. This contrasts with the view of other published studies [63], [64], that there is a differential pattern of forcing ranging from top-down in species-poor, cold water systems to bottom-up in warmer, more species-rich systems. In the cold, species-poor Barents Sea, some degree of top-down control is to be expected. However, we do not suggest that the existence of top-down effects excludes bottom-up mechanisms.

We found no significant associations of interannual variability in Chl *a* with the biomass of capelin or all pelagic fish. On the other hand, a positive Chl *a* -zooplankton association in the eastern Atlantic sector suggests a possible bottom-up effect of phytoplankton on zooplankton biomass in this region. Recently published work indicates that zooplankton biomass in the Barents Sea appears to be controlled both by fish (top-down) and climate (bottom-up) forcing [46]. The positive significant relationship between NPP in the eastern Atlantic sector and total pelagic fish biomass in the Barents Sea could be indicative for bottom-up trophic interactions. We did not observe any negative relationship between zooplankton and NPP, indicating that a potential top-down effect of fish on zooplankton may not be responsible for the increasing NPP in the system. From our results we interpret that the increase in NPP is mainly climate driven and that the fish might have benefited from increased NPP channeled through zooplankton. In other study regions, ranging from southern California to western Alaska, it has been shown that a large proportion of resident fish production is controlled by bottom-up trophic interactions [59]. In the above study based on analysis of spatial variability, high regional NPP produced generally high fishery yields, which is similar to what we observe in the eastern Atlantic sector of the Barents Sea based on the analysis of temporal variability.

Planktivorous fish in the Barents Sea do not feed only on copepods but also heavily on other organisms such as krill [65], [66]. Krill is also highly dependent on NPP because the dominant krill species in the Barents Sea, e.g. *T. inermis*, are predominantly herbivorous and depend on the spring bloom for their development [19], [20]. The impact of these organisms on NPP and interactions with their predators were not assessed in this study. In the last decade, krill abundance has been high [12], indicating good feeding conditions for these organisms. The improved/stable NPP conditions in the Barents Sea in recent years have likely led to better growth conditions for herbivorous meso- and macrozooplankton, which channelled energy to higher trophic levels.

Although the present study has its main emphasis on the importance of NPP for mesozooplankton production, it must not be forgotten that in the Barents Sea, like many other areas of world’s oceans, the microbial loop also plays an important role in the energy transfer towards higher trophic levels [67]. The role of the microbial loop may be significant, especially after the spring bloom. Unfortunately, there are no thorough field investigations that can provide a quantitative assessment of its importance in the Barents Sea. Most of the information we have at present is based on model results. Model results (not validated with field data), suggest that the microbial grazing is far greater than that of the larger *Calanus* species, and that *Calanus* need to graze both on autotrophic and heterotrophic microplankton since large-sized autotrophs can be scarce [68]. Some studies using inverse modeling also show that the microbial loop in the southern Barents Sea is not only important during summer, when protozoa fed mostly on bacteria, but also during the spring bloom when protozoa fed on bacteria as well as on phytoplankton [69]. According to their investigations, protozoa were clearly not the preferred prey of copepods during spring, but constituted about 80–90% of the copepod diet during summer. A study by Rey et al. (unpublished) in the Barents Sea seems to indicate that the regenerated primary production, which occurs mainly through the microbial loop, may be of lesser importance in the Atlantic sector of the BS (the largest region contributing to the total NPP) than previously thought.

### Summary of Ecosystem’s Response to Climate Variability

Our analyses clearly reveal that climate variability is strongest in the Arctic and eastern Atlantic sectors of the Barents Sea. The interannual variability in NPP is likely a response to climate variability. The most notable ways climate can force phytoplankton dynamics are the increase in temperature, OW, and duration of the open water season. The zooplankton biomass, and potential production, in the northern Barents Sea appears to be controlled largely through predation pressure, although in some regions, especially in areas where we observe large climate changes (e.g. ATLE), statistically positive significant linkages between Chl *a* and zooplankton were observed. Positive significant associations between NPP and fish biomass in this region is also indicative of bottom-up forcing on higher trophic levels. High pelagic fish biomass and good feeding conditions, currently observed in the Barents Sea ecosystem [5], [70], and also observed in this study is likely a positive response to changes in climate. However, longer time series will be necessary to fully understand how the changes in climate forcing will affect the dynamics of biomass and productivity in the Barents Sea.

The Barents Sea ecosystem shows a) strong effects of climate on NPP, b) strong trophic interactions, and c) potential top-down and bottom-up processes, which differ regionally and seasonally. Assessing the processes and dynamics in the Barents Sea qualitatively and quantitatively remains an important future task in order to understand and manage this ecosystem as a whole.

## Supporting Information

Figure S1 **Biomass of** ***Calanus finmarchicus*** **at the FB section estimated from observations and model simulations.**
(TIF)

Figure S2 **Large scale biomass distribution of** ***Calanus finmarchicus*** **estimated from model simulations and mesozooplankton observations.**
(TIF)

Table S1 **Pearson correlation coefficients among different variables for the whole study area of the Barents Sea.**
(DOC)

Table S2 **Pearson correlation coefficients for the Arctic region of the Barents Sea.**
(DOC)

Table S3 **Pearson correlation coefficients for the Atlantic east of the Barents Sea.**
(DOC)

Table S4 **Pearson correlation coefficients for the Atlantic west of the Barents Sea.**
(DOC)

Table S5 **Pearson correlation coefficients for the coastal east of the Barents Sea.**
(DOC)

Table S6 **Pearson correlation coefficients for the coastal west of the Barents Sea.**
(DOC)

Text S1 **Model description and validation.**
(DOC)
