# Supplementary figures and images for: Productivity in the Barents Sea - Response to Recent Climate Variability
Source: PLoS One. 2014 May 1;9(5):e95273. doi: 10.1371/journal.pone.0095273 (PMC4006807; doi:10.1371/journal.pone.0095273)

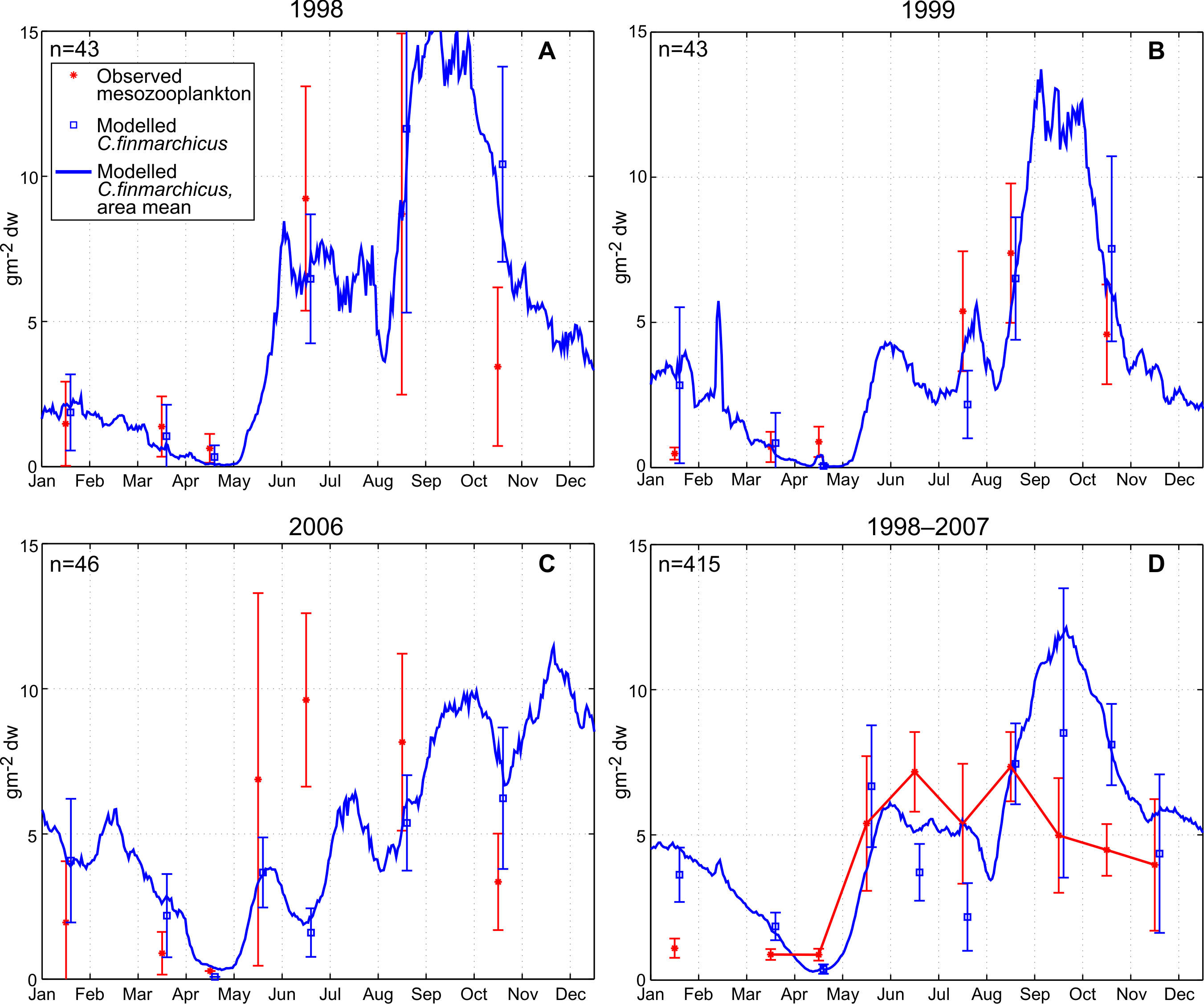

Supplement: Figure S1 — Biomass of Calanus finmarchicus at the FB section estimated from observations and model simulations. (TIF) [file pone.0095273.s001.tif]

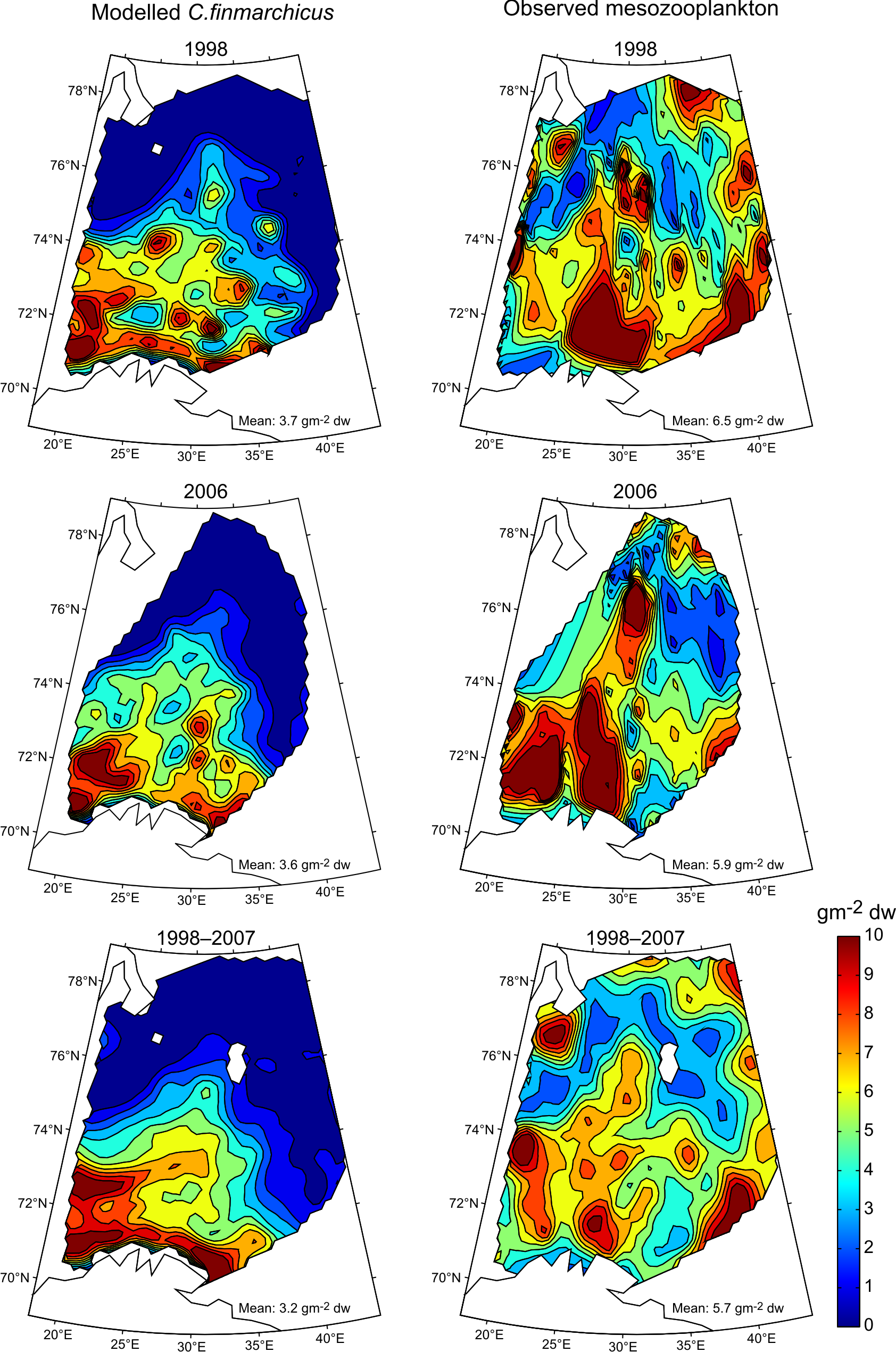

Supplement: Figure S2 — Large scale biomass distribution of Calanus finmarchicus estimated from model simulations and mesozooplankton observations. (TIF) [file pone.0095273.s002.tif]
