# Supplementary material for: Productivity in the Barents Sea - Response to Recent Climate Variability
Source: PLoS One. 2014 May 1;9(5):e95273. doi: 10.1371/journal.pone.0095273 (PMC4006807; doi:10.1371/journal.pone.0095273)
Supplement: Table S1 — Pearson correlation coefficients among different variables for the whole study area of the Barents Sea. (DOC) [file pone.0095273.s003.doc]

**Table S1:** Pearson correlation coefficients among different variables for the **whole study area** of the Barents Sea.

| **Area: Barents Sea** | Sat Chl *a* | Production | NPP | OW | ZB >2000µm | ZB 1000-2000µm | ZB 1000-180µm | ZB sum | Kola temperature | Capelin | Total pelagic fish |
| --- | --- | --- | --- | --- | --- | --- | --- | --- | --- | --- | --- |
| Year |  |  | *0.52* | **0.61*** | *-0.48* |  | **-0.75**** | *-0.48* | **0.56*** |  |  |
| Sat Chl *a* |  | **0.96***** | **0.80**** |  |  |  |  |  |  |  |  |
| Production |  |  | **0.80**** |  |  |  |  |  |  |  |  |
| NPP |  |  |  | *0.49* |  |  |  |  |  |  | *0.47* |
| OW |  |  |  |  | *-0.49* |  |  |  | **0.84**** |  | *0.46* |
| ZB >2000µm |  |  |  |  |  | *0.43* |  | **0.85**** |  | *-0.56* |  |
| ZB 1000-2000µm |  |  |  |  |  |  |  | **0.68*** |  | *-0.56* |  |
| ZB 1000-180µm |  |  |  |  |  |  |  | *0.43* |  |  |  |
| ZB sum |  |  |  |  |  |  |  |  |  | *-0.61* |  |
| Kola temperature |  |  |  |  |  |  |  |  |  |  | **0.48*** |
| Capelin |  |  |  |  |  |  |  |  |  |  |  |

Mean (1998-2011) values for satellite-derived chlorophyll (Sat Chl *a*, mg m^-3^), production per unit area (g C m^-2^ year^-1^), net primary production (NPP, Tg C year^-1^), open water area (OW, km^2^), mesozooplankton biomass (ZB, g m^-2^) of different size fractions, sum of mesozooplankton biomass (ZB, g m^-2^), Kola temperature (°C), and capelin and total pelagic fish stock (million tonnes).

Correlation coefficients <0.4 are not shown. Significance levels taking autocorrelation into account (except for row ‘Year’): *** p<0.001, ** p<0.01, * p<=0.05, (*) p<0.1. Kola temperature, capelin biomass and total pelagic fish biomass are aggregated values for the whole study area.
