# Supplementary material for: Productivity in the Barents Sea - Response to Recent Climate Variability
Source: PLoS One. 2014 May 1;9(5):e95273. doi: 10.1371/journal.pone.0095273 (PMC4006807; doi:10.1371/journal.pone.0095273)
Supplement: Table S3 — Pearson correlation coefficients for the Atlantic east of the Barents Sea. (DOC) [file pone.0095273.s005.doc]

**Table S3:** Pearson correlation coefficients for the **Atlantic east** of the Barents Sea (see Table S1 for further explanations).

| **Region:**  **Atlantic east** | Sat Chl *a* | Production | NPP | OW | ZB >2000µm | ZB 1000-2000µm | ZB 1000-180µm | ZB sum | Kola temperature | Capelin | Total pelagic fish |
| --- | --- | --- | --- | --- | --- | --- | --- | --- | --- | --- | --- |
| Year |  |  | **0.54*** | **0.60*** |  |  | **-0.70**** | **-0.57*** | **0.56*** |  |  |
| Sat Chl *a* |  | **0.86***** |  | *-0.53* | **0.72**** | *0.53* |  | **0.69**** | **-0.62*** |  |  |
| Production |  |  | **0.56*** |  | **0.56*** |  |  | **0.49*** |  |  |  |
| NPP |  |  |  | **0.70*** |  |  |  |  | *0.48* |  | **0.66*** |
| OW |  |  |  |  | **-0.72*** | *-0.51* |  | **-0.59*** | **0.79**** |  | 0.57(*) |
| ZB >2000µm |  |  |  |  |  | **0.72*** |  | **0.77**** | *-0.45* | -0.54(*) |  |
| ZB 1000-2000µm |  |  |  |  |  |  |  | **0.84***** |  | -0.60(*) |  |
| ZB 1000-180µm |  |  |  |  |  |  |  | *0.46* |  |  |  |
| ZB sum |  |  |  |  |  |  |  |  | *-0.46* | *-0.52* |  |
| Kola temperature |  |  |  |  |  |  |  |  |  |  | **0.48*** |
| Capelin |  |  |  |  |  |  |  |  |  |  |  |
