# Supplementary material for: Productivity in the Barents Sea - Response to Recent Climate Variability
Source: PLoS One. 2014 May 1;9(5):e95273. doi: 10.1371/journal.pone.0095273 (PMC4006807; doi:10.1371/journal.pone.0095273)
Supplement: Table S5 — Pearson correlation coefficients for the coastal east of the Barents Sea. (DOC) [file pone.0095273.s007.doc]

**Table S5:** Pearson correlation coefficients for the **coastal east** of the Barents Sea (see Table S1 for further explanations).

| **Region:**  **Coastal east** | Sat Chl *a* | Production | NPP | OW | ZB >2000µm | ZB 1000-2000µm | ZB 1000-180µm | ZB sum | Kola temperature | Capelin | Total pelagic fish |
| --- | --- | --- | --- | --- | --- | --- | --- | --- | --- | --- | --- |
| Year |  |  |  | **0.55*** |  |  | *-0.52* |  | **0.56*** |  |  |
| Sat Chl *a* |  | **0.93***** | **0.84***** |  |  |  |  |  |  |  |  |
| Production |  |  | **0.96***** |  |  |  |  |  |  |  |  |
| NPP |  |  |  | *0.51* |  |  |  |  |  |  |  |
| OW |  |  |  |  |  |  | *-0.56* |  | 0.60(*) |  |  |
| ZB >2000µm |  |  |  |  |  | *0.54* | *0.41* | **0.91**** |  |  |  |
| ZB 1000-2000µm |  |  |  |  |  |  | *0.55* | **0.81*** |  |  |  |
| ZB 1000-180µm |  |  |  |  |  |  |  | **0.67*** | *-0.55* | *0.53* |  |
| ZB sum |  |  |  |  |  |  |  |  |  |  |  |
| Kola temperature |  |  |  |  |  |  |  |  |  |  | **0.48*** |
| Capelin |  |  |  |  |  |  |  |  |  |  |  |
