# Supplementary material for: Productivity in the Barents Sea - Response to Recent Climate Variability
Source: PLoS One. 2014 May 1;9(5):e95273. doi: 10.1371/journal.pone.0095273 (PMC4006807; doi:10.1371/journal.pone.0095273)
Supplement: Text S1 — Model description and validation. (DOC) [file pone.0095273.s009.doc]

**Text S1: Model description and validation**

The modeling system norwecom.e2e [27-29] consists of a full 3-dimensional ocean model and the biogeochemical model NORWECOM that is two-way coupled to an individual-based model for *C. finmarchicus*. The model has been run in offline mode using physical fields (velocities, salinity, temperature, water level, and ice) from the Regional Ocean Model System (ROMS). The horizontal grid is a stretched spherical coordinate grid that covers the North Atlantic from 20 degrees south towards the Arctic, with a resolution of approximately 20x20 km, while the vertical dimension is resolved in 20 bottom following sigma layers, stretched to increase vertical resolution near the surface and bottom. A nutrient-phytoplankton model is coupled to the physical model through the subsurface light, the hydrography, and the horizontal and vertical movement of water masses. The modeled processes are primary production, respiration, algae death, re-mineralization of inorganic nutrients from dead organic matter, self shading, turbidity, sedimentation, re-suspension, sediment burial, and de-nitrification. The individual-based model for *C. finmarchicus* is coupled to the nutrient-phytoplankton model and enforces grazing on the phytoplankton. It takes into account growth, mortality, movement, and reproduction of *C. finmarchicus* as well as adaptive traits, which control the interaction with the environment.

The initial distribution fields for *C. finmarchicus* are based on an overwintering population of *C. finmarchicus* distributed in the deeper Norwegian Sea basins and also in the Greenland and Barents Sea, evolved through a four year long adaptation process. Estimates of *C. finmarchicus* biomass and production for different seasons and years were generated as per unit carbon and converted to dry weight using a conversion factor of two [S1]. For comparison with observations, model biomass values were picked that are as close to the time and position of observations as possible, and were thereafter grouped into monthly (FB section) or autumn means (whole study area). The model simulation ends in 2007 due to limited ocean forcing fields and therefore, does not cover the whole period of remote-sensing and observed data (1998-2011).

Modeled zooplankton biomass for the FB section (whole year), and the whole Barents Sea, (autumn) in the overlapping time period (1998-2007) was validated using observed mesozooplankton biomass data (Figs. S1 and S2, respectively). The variability between years in the FB section is large. The period mean of modeled biomass was close to observations in spring, but overestimated the observed biomass by a factor of two in autumn months (Fig. S1). The reason for this could be that *C. finmarchicus* in this model setup has no competition from other phytoplankton grazers, and also has a simplified constant predation rate which may allow unrealistic high population growth. For the autumn mean biomass distribution, the model reproduces the *in situ* spatial pattern with high biomass values in the Atlantic sector, but underestimates mesozoplankton biomass in the northern and eastern regions (Fig. S2). However, both the modeled and observed values show some interannual variability in spatial patterns, as shown exemplarily for two selected years (1998 and 2006, Fig. S2). From the modeled and observed autumn biomass area means of this study, the proportion of modeled *C. finmarchicus* could constitute 57%, 61%, and 56% of the observed mesozooplankton, respectively for the autumn 1998 and 2006 and the 1998-2007 average.

References

S1 Hirche HJ, Brey T, Niehoff B (2001) A high-frequency time series at ocean Weather ship station M (Norwegian Sea): population dynamics of *Calanus finmarchicus*. Mar Ecol Progr Ser 219: 205-219.

Figure S1*. Calanus finmarchicus* biomass (g m^-2^ dw) at the FB section estimated from observations and model simulations from 1998, 1999, 2006, and composite for the years 1998-2007. Observed mesozooplankton biomass (red circles) of the size classes <1000µm and 1000-2000µm is assumed to be predominantly *C. finmarchicus*. Modeled *C. finmarchicus* biomass is shown as I) monthly means of values in positions picked at the same time and horizontal and vertical position as observations are available (blue squares), and II) daily values in the complete Fugløya-Bjørnøya section (blue line). Standard errors are indicated by vertical lines and n is the number of observations.

Figure S2. August-October large-scale biomass (g m^-2^ dw) distribution of *Calanus finmarchicus* estimated from model simulations and mesozooplankton observations. The average biomasses in two selected years (1998 and 2006) and over the composite years 1998-2007 are presented. Grand means of modeled *C. finmarchicus* biomass and observed mesozooplankton biomass within the whole area are given lower right of each panel. The observed mean biomass values were equally weighted and interpolated to the nearest grid point by Delaunay triangulation of the data.
